# Functional Outcomes and Complications After Salvage Total Laryngectomy for Residual, Recurrent, and Second Primary Squamous Cell Carcinoma of the Larynx and Hypopharynx: A Multicenter Retrospective Cohort Study

**DOI:** 10.3389/fonc.2020.01390

**Published:** 2020-09-02

**Authors:** Jeroen Meulemans, Hannelore Demarsin, Jens Debacker, Gaël Batailde, Tillo Mennes, Annouschka Laenen, Ann Goeleven, Peter Neyt, Christophe Vanclooster, Tom Vauterin, Pierre Delaere, Wouter Huvenne, Vincent Vander Poorten

**Affiliations:** ^1^Otorhinolaryngology-Head and Neck Surgery, University Hospital Leuven, Leuven, Belgium; ^2^Department of Oncology, Section Head and Neck Oncology, KU Leuven, Leuven, Belgium; ^3^Department of Head and Skin, Ghent University, Ghent, Belgium; ^4^Otorhinolaryngology-Head and Neck Surgery, AZ Sint-Jan, Bruges, Belgium; ^5^Interuniversity Center for Biostatistics and Statistical Bioinformatics, Leuven, Belgium; ^6^Otorhinolaryngology-Head and Neck Surgery, Swallowing Clinic, University Hospital Leuven, Leuven, Belgium; ^7^Otorhinolaryngology-Head and Neck Surgery, AZ Sint-Lucas, Ghent, Belgium; ^8^Otorhinolaryngology-Head and Neck Surgery, University Hospital Ghent, Ghent, Belgium

**Keywords:** complications, functional outcomes, hypopharynx, larynx, recurrence, salvage surgery, squamous cell carcinoma, total laryngectomy

## Abstract

**Background/Purpose:** We analyzed complications and functional outcomes and aimed at identifying prognostic factors for functional outcomes and complications in patients who underwent salvage total laryngectomy (STL) for residual, recurrent, and second primary squamous cell carcinoma (SCC) of the larynx and hypopharynx after initial (chemo)radiation.

**Methods:** Retrospective cohort study of patients who underwent STL in four major Belgian reference hospitals between 2002 and 2018. Prognostic factors for functional outcomes and complications were identified with uni- and multivariable analysis.

**Results:** A total of 405 patients were included in the final analysis. STL was performed for residual tumor (40.2%), local recurrence (40.5%), or second primary laryngeal or hypopharyngeal SCC (19.4%). Early postoperative complications were experienced by 34.2% of patients: postoperative hemorrhage occurred in 5.4%, wound infection in 16.2%, and clinical pharyngocutaneous fistula (PCF) in 25.5% of patients. Early readmission proved necessary in 15.1% of cases, most often due to late PCF development (72.2%). Patients achieved total peroral intake in 94.2% of cases. However, subjective dysphagia was reported by 31.3% of patients during follow-up. Functional speech, defined as functional communication by speech without additional aids, was reported in 86.7% of cases and was most often achieved by tracheo-esophageal puncture (TEP) (94.1%). In a multivariable model, lower preoperative hemoglobin (<12.5 g/dl) was identified as an independent prognostic factor for higher overall complication rate. No risk factors were found significant for clinical fistula formation. Vascularized tissue augmentation did not significantly prevent clinical PCF. Patients with positive section margins, patients initially treated with surgery combined with adjuvant RT (vs. radiotherapy alone), and those developing PCF after STL were less likely to achieve total peroral intake. Postoperative dysphagia proved more likely in patients who developed a PCF postoperatively, and less likely in patients who underwent STL without partial pharyngectomy and in patients with myocutaneous pectoralis major (PM) flap reconstruction, compared to muscle onlay PM flap. Achieving postoperative functional speech proved most likely in patients with smaller tumors (lower pT classification) and free section margins.

**Conclusion:** Substantial complication rates and favorable functional outcomes are reported after STL.

## Introduction

The management of advanced hypopharyngeal and laryngeal cancers has seen drastic changes over the last decades. The Veterans Affairs (VA) trial in 1991 followed by the Radiation Therapy Oncology Group (RTOG) 91–11 trial in 2003 showed similar survival rates between organ preservation therapy and total laryngectomy (TL) for advanced laryngeal cancers (1–3). These comparable oncologic outcomes, combined with a high probability of preserving speech and swallowing functions, made radiotherapy (RT), and chemoradiotherapy (CRT) the standard of care to treat laryngeal and hypopharyngeal cancers in many institutions (4–7). However, on the long term, radiation-based therapy also has the potential to compromise these functions due to permanent tissue damage (RT-induced fibrosis and xerostomia) (8). In 21–66% of patients who are primarily treated with organ preservation strategies, salvage total laryngectomy (STL) eventually proves necessary when tumor recurrence occurs or residual cancer is diagnosed following RT/CRT (9, 10). However, STL carries a higher complication risk when compared to primary laryngectomy in a non-irradiated patient. Radiation affects the tissue's microvasculature; more precisely, it causes subintimal fibrosis, endarteritis, and thrombus formation, inducing a hypovascular, hypocellular, and hypoxic environment, which in turn results in worse healing conditions (11). Pharyngocutaneous fistula (PCF), an abnormal communication between the digestive tract and the cervical skin or tracheostoma resulting in salivary leakage, is the most common major wound complication after STL (12–14). PCF is associated with prolonged hospitalization, the potential need for surgical revisions, and it delays initiation of peroral feeding and (re-)irradiation when indicated. Moreover, PCF can potentially lead to death due to carotid blowout (12–14). A recent systematic review of 49 studies investigating complications following STL reported an overall complication rate of 67.5%, with a pooled incidence of 28.9% (95% CI 25.5–32.5%) of patients developing a PCF (14). Multiple risk factors have been linked with a higher PCF and complication rate after STL, but results are not consistent across studies (14). Factors reported to predispose to PCF include preoperative (chemo)radiotherapy, types of surgery, neck dissection, pharyngeal closure techniques, positive tumor margins, presence of a preoperative tracheotomy, comorbidities, tumor site and stage, nutrition status, and hemoglobin levels (14–16). The aim of this multicenter retrospective cohort study is to report and analyze functional outcomes and complications after STL and identify prognostic factors for functional outcome and complications.

## Patients and Methods

### Ethical Considerations

The current study was approved by and carried out in accordance with the recommendations of the Coordinating Institutional Review Board (University Hospital Leuven Committee for Medical Ethics) and the local Ethical boards of participating centers. Informed consent was waived given the retrospective nature of this study (study registration numbers S61749 and B670201938931).

### Patients

We conducted a multicentric retrospective cohort study of all patients who underwent STL for squamous cell carcinoma (SCC) of the larynx or hypopharynx in a 16-year period from 01.01.2002 to 31.12.2018. Hospitals who participated in this study were two academic centers (UZ Leuven, and UZ Ghent) and two major regional centers (AZ Sint-Jan in Bruges, AZ Sint-Lucas in Ghent). A total of 405 patients were identified and included in this retrospective review upon screening of hospital databases.

Predefined inclusion criteria were as follows:

– STL with or without partial pharyngectomy for local recurrent (diagnosis >12 months and <60 months after primary treatment) or residual (diagnosis <12 months after primary treatment) SCC of a laryngeal or hypopharyngeal tumor primarily treated with CRT or RT.
– STL with or without partial pharyngectomy for second primary SCC in an irradiated larynx or hypopharynx after primary treatment with RT or CRT. According to the criteria of Warren and Gates and its modification by the National Cancer Institute, second primary SCC was defined as a metachronous or synchronous tumor evolving from a different anatomical subsite as the primary tumor, or as a synchronous tumor in the same subsite as the primary tumor but developing later than 60 months after diagnosis of the primary tumor (17–19). As such, local recurrence of a laryngeal/hypopharyngeal tumor after initial (chemo)radiation was defined as malignancy in the same laryngeal/hypopharyngeal subsite as the primary tumor diagnosed within 60 months after the diagnosis of the first malignancy.

Predefined exclusion criteria were as follows:

– STL performed for non-squamous cell carcinomas
– Patients who underwent STL with circumferential (total) pharyngectomy requiring reconstruction with free jejunal transfer, gastric pull-up procedure (gastric transposition), colon interposition, or tubed/tunneled(myo)cutaneous flaps (e.g., anterolateral thigh, radial forearm flap, latissimus dorsi, myocutaneous pectoralis major)
– Patients who underwent a “functional” laryngectomy [e.g., for persistent aspiration after primary radio(chemo)therapy without evidence for recurrent or residual malignancy]

### Data

The medical records of 405 patients were retrospectively reviewed. The data related to patient, tumor, and treatment and complications as well as functional outcomes were retrieved from the patient's health records. All data were pseudonymized in the participating centers and eventually gathered in one central database with the University Hospitals Leuven as data-controller. Tumor staging was reviewed according to the most recent 8th edition of the UICC/AJCC TNM classification. The reference limits of biomarkers were chosen according to limits suggested in previous studies and general accepted normality ranges, in which anemia was defined as hemoglobin (Hb) <12.5 g/dl and hypoalbuminemia as albumin (Ab) <3.5 g/dl (16, 20–22). PCF was defined as an abnormal communication between the digestive tract/neopharynx and the cervical skin and/or tracheostoma resulting in a clinically visible salivary leakage. Postoperative hemorrhage was defined as postoperative bleeding that required surgical revision. Postoperative wound infection was defined as a local wound infection with or without abcedation that required the administration of antibiotics outside of the routine postoperative antibiotics administration and/or surgical drainage. Various variables were assessed as potential prognostic factors for complication rate, as well as for “overall complication rate” as for PCF specifically (Table 1). We also examined the relationship between the results of the postoperative upper gastrointestinal tract radiograph (esophagram/esophageal fluoroscopy) with aqueous low osmolar non-ionic iodine contrast (Ultravist® or iopromide) and clinical PCF formation. Functional outcomes assessed were time to resume oral feeding, achievement of total peroral intake, achievement of functional speech, presence (or absence) of postoperative permanent tube feeding, and postoperative dysphagia. Functional speech was defined as functional communication by speech [esophageal speech, electrolarynx speech, and trachea-esophageal puncture (TEP) speech] without the need for additional aids (e.g., writing, gestures, etc.). Dysphagia was defined as a sense of obstruction while swallowing with/without signs of regurgitation, mentioned in medical files, with or without objective confirmation of a neopharyngeal stenosis on esophagram or gastroscopy/esophagoscopy. Variables analyzed as potential risk factors for functional outcomes were type of salvage operation (TL vs. TL with partial pharyngectomy), reconstruction type [pectoralis major (PM) flap muscle onlay vs. myocutaneous PM flap vs. none], treatment type of the first tumor (surgery and adjuvant RT vs. surgery and adjuvant CRT vs. definitive CRT vs. definitive RT), type of secondary tumor (local recurrence vs. residual tumor vs. second primary), total RT dose at the level of the larynx during previous treatment, tracheo-esophageal puncture (primary vs. secondary vs. none), postoperative complications (any type), postoperative clinical PCF formation, adjuvant treatment after STL, and margin status (free vs. close vs. positive).

**Table 1:** Factors associated with overall complications and PCF formation (univariable).

**Variables**	**Postoperative complications**			**PCF**		
	**OR**	**95% CI**	**(*p*)**	**OR**	**95% CI**	**(*p*)**
Preoperative Hb ≥12.5 g/dl vs. <12.5 g/dl	0.579	0.360–0.931	**(0.0241)**	0.820	0.489–1.373	(0.4495)
Postoperative Hb ≥12.5 g/dl vs. <12.5 g/dl	0.442	0.241–0.812	**(0.0085)**	0.516	0.263–1.014	(0.0550)
Preoperative albumin						
≥3.5 g/dl vs. <3.5 g/dl	1.584	0.654–3.837	(0.3079)	1.062	0.419–2.693	(0.8999)
Postoperative albumin						
≥3.5 g/dl vs. <3.5 g/dl	0.630	0.259–1.533	(0.3090)	0.358	0.115–1.117	(0.0769)
Preoperative tracheotomy						
Yes vs. No	1.868	0.942–3.706	(0.0737)	1.896	0.931–3.864	(0.0780)
Preoperative active smoking						
Yes vs. No	0.907	0.556–1.479	(0.6957)	0.960	0.561–1.644	(0.8824)
Type of salvage operation						
STL+partial pharyngectomy vs. STL	1.506	0.958–2.369	(0.0761)	1.147	0.707–1.861	(0.5793)
Reconstruction type						
PM flap muscle onlay vs. myocutaneous PM flap	0.678	0.388–1.182	(0.1705)	0.902	0.486–1.674	(0.7440)
PM flap muscle onlay vs. none	0.895	0.546–1.467	(0.6596)	0.800	0.470–1.363	(0.4116)
myocutaneous PM flap vs. none	1.321	0.783–2.228	(0.2971)	0.887	0.499–1.576	(0.6827)
Type of second tumor						
Local recurrence vs. Residual tumor	0.841	0.526–1.347	(0.4721)	0.618	0.368–1.038	(0.0687)
Local recurrence vs. Second primary	0.778	0.437–1.383	(0.3921)	0.717	0.377–1.361	(0.3091)
Residual tumor vs. Second primary	0.924	0.523–1.634	(0.7868)	1.160	0.627–2.147	(0.6357)
Treatment initial tumor (residual/recurrent)^*^						
Surgery + adjuvant RT vs. definitive CRT	0.873	0.208–3.670	(0.8527)	1.829	0.426–7.851	(0.4169)
Surgery + adjuvant RT vs. definitive RT	1.813	0.474–6.943	(0.3849)	2.522	0.656–9.699	(0.1783)
Surgery + adjuvant RT vs. surgery + adjuvant CRT	2.400	0.175–32.868	(0.5122)			
Definitive CRT vs. definitive RT	2.078	1.097– 3.937	**(0.0249)**	1.379	0.690–2.758	(0.3631)
Definitive CRT vs. surgery + adjuvant CRT	2.749	0.266–28.423	(0.3961)			
Definitive RT vs. surgery + adjuvant CRT	1.323	0.135–12.927	(0.8097)			
Treatment initial tumor (second primary)^*^						
Surgery + adjuvant RT vs. definitive CRT	0.563	0.090–3.518	(0.5385)	0.562	0.090–3.518	(0.5385)
Surgery + adjuvant RT vs. definitive RT	0.619	0.147–2.608	(0.5130)	1.281	0.293–5.598	(0.7418)
Definitive CRT vs. definitive RT	1.100	0.276–4.380	(0.8925)	2.278	0.551–9.417	(0.2556)
Radiotherapy initial tumor dose on larynx (Gray)						
+1 unit	0.993	0.960–1.026	(0.6632)	0.982	0.948–1.017	(0.3032)
Extent of neck dissection						
Bilateral vs. Ipsilateral	0.754	0.458–1.240	(0.2653)	0.597	0.342–1.043	(0.0699)
Bilateral vs. None	1.001	0.591–1.695	(0.9975)	0.651	0.364–1.162	(0.1466)
Ipsilateral vs. None	1.328	0.786–2.243	(0.2888)	1.089	0.627–1.892	(0.7620)
Tracheo-oesofageal puncture						
Secondary/none vs. Primary	1.505	0.800–2.831	(0.2053)	1.463	0.749–2.858	(0.2658)
Treating center, global test	-	-	**(0.0463)**	-	-	(0.1640)
Treating center, global test, after correction for confounders	-	-	(0.1904)	-	-	(0.1821)

Continuous variables: OR > (<)1: increased (decreased) risk with increasing variable level.

Binary variables/pairwise tests: OR > (<)1: higher (lower) risk for first category. Reconstruction type: radial forearm free flap (n = 3) and other (n = 1) excluded.

* Not all pairwise comparisons were estimable due to small groups/zero events.

*Bold values are statistically significant (p < 0.05)*.

### Treatment

STL was performed in all centers according to a standardized and well-described surgical technique (23). Surgical details differed between centers: after resection of the larynx or laryngopharynx, a myotomy of the cricopharyngeal muscle was performed in all cases; pharyngeal closure was performed with interrupted or running (depending on the center) absorbable sutures in a T-fashion in two layers (mucosa and submucosa) or three layers (mucosa, submucosa, and constrictor muscle, depending on the center); neurectomy of the pharyngeal plexus was omitted. The tracheostoma was created by suturing the cartilaginous trachea to the caudal skin flap, while the membranous trachea was sutured to the cranial skin flap. Neck dissections were always performed when a cN+ neck was present. However, the decisions to perform elective neck dissections of the lateral compartment, thyroidectomy (non, partial or complete), and reconstruction (PM onlay, PM myocutaneous inset or primary closure) were left at the surgeon's discretion and depended on institutional practice. Central neck dissection was always performed and varied from a formal bilateral level VI and VII dissection to more limited dissection of trachea-esophageal nodes, depending on the institution, surgeon, and pathology. All patients received per- and postoperatively intravenous prophylactic antibiotic treatment with cephazolin + metronidazole or amoxicillin + clavulanic acid for 2 days. On the 10th postoperative day, all patients underwent an esophagram, except when a clinical PCF was present. If the esophagram did not show signs of PCF formations, peroral feeding was initiated the following day with liquid and semiliquid foods. In the case of a clinical PCF or an esophagram showing (subclinical) fistula, peroral feeding was postponed until subsequent esophagrams (5–7 days after the prior) suggested complete healing of the PCF tract. Most frequently, PCFs were managed conservatively (nil per os, cuffed tracheostomy tube in case of PCF draining in the tracheostoma). Surgical interventions to close the PCF (e.g., with PM of free flaps) were considered when conservative measures failed or when fistula trajectories were judged unlikely to heal with conservative measures alone, or when evolution to serious complications (e.g., carotid blowout) was suspected. Postoperative care, including feeding management, antibiotics, and imaging, were uniform among centers. Adjuvant therapy (e.g., re-irradiation) after STL was performed according to the consensus of the multidisciplinary hospital's tumor board.

### Statistical Analysis

Logistic regression models were used to analyze prognostic effects of patient or treatment characteristics on binary outcomes, including complications or functional outcomes and re-admission. Results are presented as odds ratios (OR) with 95% confidence intervals. A forward selection procedure was used for the selection of a multivariable model of independent prognostic variables for complications or functional outcomes, with a 5% significance level for entering variables. Linear regression models were used to analyze prognostic effects of patient or treatment characteristics on hospital stay. Analyses were performed on a log-transformed outcome variable. Results are presented as relative effects (RE) with 95% confidence intervals. A backward selection procedure was used for the selection of a multivariable model of independent prognostic variables for hospital stay, with a 5% significance level for removal of variables. All tests are two-sided, and a 5% significance level was assumed for all tests. No corrections for multiplicity were performed due to the explorative nature of the study. Analyses have been performed using SAS software (version 9.4 of the SAS System for Windows). Follow-up summary statistics were based on the Kaplan–Meier estimate of potential follow-up (24).

## Results

### Patient Characteristics

This cohort study included 405 patients, consisting of 378 males (93.3%) and 27 females (6.7%), with a mean age of 65.3 years (SD 9.8 years, range 40.3–91.2 years). At the time of diagnosis of the initial tumor, 76.9 % (*n* = 279) and 29.0% (*n* = 99) of patients were active smokers and active heavy drinkers, respectively. A substantial amount of patients (*n* = 106, 30.6%) still smoked at the time of diagnosis of the recurrent/residual/second primary tumor. Thirty-eight patients (10.4%) were tracheotomy-dependent before surgery. The complete patient characteristics are summarized in Table 2.

**Table 2:** Patient characteristics.

**Patient characteristics**	***n***	**%**
**Gender (*****n*** **=** **405)**		
Male	378	93.33
Female	27	6.67
**Age at time of diagnosis recurrent/second primary tumor**		
*N*	395	
Mean	65.31	
Std	9.809	
Median	65.37	
IQR	(57.82–71.92)	
Range	(40.28–91.24)	
**Preoperative active smoking (*****n*** **=** **347)**		
No	241	69.45
Yes	106	30.55
**Active smoking at time of diagnosis (*****n*** **=** **363) at the time of diagnosis of the initial tumor**		
No	84	23.14
Yes	279	76.86
**Active drinking at time of diagnosis (*****n*** **=** **341) at the time of diagnosis of the initial tumor**		
No	242	70.97
Yes	99	29.03
**Preoperative tracheotomy (*****n*** **=** **366)**		
No	328	89.62
Yes	38	10.38

### Tumor Characteristics

Initial tumor characteristics are shown in Table 3. Primary tumors were almost exclusively squamous cell carcinomas (99.3%) and were most frequently located in the glottis (*n* = 260; 64.7%). Most initial tumors were considered as locoregionally limited disease (cT1 and cT2 in 41.4 and 31.4% of cases, cN0 in 81.6% of cases). Stage I (*n* = 160; 39.9%) was the most common overall stage. In this group of primary tumors, 308 patients (76.4%) were treated with RT alone. Table 4 shows secondary tumor characteristics. Mean time between treatment of the first tumor and the diagnosis of the second tumor was 2.7 years (SD 4.1 years, range 0.1–35.5 years). The second tumor that necessitated STL was considered a residual tumor after organ preserving treatment in 40.2% of cases (*n* = 162), local recurrence of the initial tumor in 40.5% of cases (*n* = 163), and a second primary carcinoma in 19.4% of cases (*n* = 78). The glottis was, again, the most commonly involved subsite (43.5%). When compared to the primary tumors, a shift is observed from locally limited disease for the initial tumor to locally advanced disease for the second tumor (cT3 and cT4a in 35.9 and 26.8% of cases, respectively). The majority of second tumors presented without nodal involvement (cN0 in 84.8% of cases).

**Table 3:** Initial tumor characteristics.

**Initial tumor characteristics**	***n***	**%**
**Histology (*****n*** **=** **398)**		
Non-squamous cell carcinoma	3	0.75
Squamous cell carcinoma	395	99.25
**Clinical tumor classification (*****n*** **=** **401)**		
Tx	2	0.50
T1	166	41.40
T2	126	31.42
T3	87	21.70
T4a	19	4.74
T4b	1	0.25
**Clinical nodal classification (*****n*** **=** **401)**		
N0	325	81.05
N1	30	7.48
N2 (no further specification)	5	1.25
N2a	7	1.75
N2b	19	4.74
N2c	15	3.74
**Clinical metastasis classification (*****n*** **=** **401)**		
M0	401	100
**Overall stage (*****n*** **=** **401)**		
I	160	39.90
II	103	25.69
III	86	21.45
IVa	51	12.72
IVb	1	0.25
**Location (*****n*** **=** **402)**		
Oropharynx	18	4.48
Hypopharynx	19	4.73
Supraglottic	96	23.88
Glottic	260	64.68
Subglottic	6	1.49
Other head neck site	3	0.75
**Treatment initial tumor (*****n*** **=** **403)**		
Surgery + adjuvant RT	22	5.46
Surgery + adjuvant CRT	6	1.49
Definitive RT	308	76.43
Definitive CRT	57	14.14
Induction chemotherapy + definitive CRT	3	0.74
Induction chemotherapy + definitive RT	1	0.25
Other	6	1.49
**RT: total dose larynx (in Gray)**		
*N*	325	
Mean	66.40	
Std	6.715	
Median	69.50	
IQR	(66.00–77.00)	
Range	(0.00–75.80)	
**RT: total dose initial tumorsite (in Gray)**		
*N*	171	
Mean	67.53	
Std	3.502	
Median	69.12	
IQR	(66.00–70.00)	
Range	(50.00–75.80)	

**Table 4:** Secondary tumor characteristics.

**Secondary tumor characteristics**	***n***	**%**
**Years prior to second tumor**		
*N*	385	
Mean	2.70	
Std	4.070	
Median	1.20	
IQR	(0.61–2.96)	
Range	(0.06–35.48)	
**Type of second tumor (*****n*** **=** **403)**		
Local recurrence	163	40.45
Second primary	78	19.35
Residual tumor	162	40.20
**Initial location second tumor (*****n*** **=** **402)**		
Hypopharynx	24	5.97
Glottic	175	43.53
Supraglottic	119	29.60
Subglottic	22	5.47
Transglottic	49	12.19
Combination	13	3.23
**Clinical tumor classification (*****n*** **=** **373)**		
Tx	2	0.54
T1	19	5.09
T1a	11	2.95
T1b	15	4.02
T2	89	23.86
T3	134	35.92
T4a	100	26.81
T4b	3	0.80
**Clinical nodal classification (*****n*** **=** **376)**		
N0	319	84.84
N1	25	6.65
N2a	2	0.53
N2b	14	3.72
N2c	10	2.66
N3	6	1.60
**Clinical metastasis classification (*****n*** **=** **376)**		
M0	374	99.47
M1	2	0.53
**Pathological tumor classification (*****n*** **=** **388)**		
Tx	3	0.77
T1	19	4.90
T1a	11	2.84
T1b	17	4.38
T2	75	19.33
T3	123	31.70
T4a	135	34.79
T4b	5	1.29
**Pathological nodal classification (*****n*** **=** **391)**		
N0	332	84.91
N1	22	5.63
N2a	4	1.02
N2b	13	3.32
N2c	9	2.30
N3	11	2.81
**Overall stage (*****n*** **=** **373)**		
I	33	8.85
II	79	21.18
III	137	36.73
IVa	113	30.29
IVb	8	2.14
IVc	3	0.80
**Section margins (*****n*** **=** **392)**		
Free margins (>5 mm)	317	80.87
Close margin (<5 mm)	45	11.48
Positive margin (<5 mm)	30	7.65

Recurrent/residual/second primary tumors were clinically staged as stage I (*n* = 33; 8.9%), II (*n* = 79; 21.2%), III (*n* = 137; 36.7%), and IV (*n* = 124; 33.2%). Postoperatively, patients were most commonly pathologically classified as pT4a (34.8%) and pN0 (84.9%). Free margins (>5 mm) were obtained after STL in 81% (*n* = 317) of patients.

### Treatment Characteristics and Complications

Details related to the surgical procedure performed are illustrated in Table 5. One hundred forty-five patients (35.9%) underwent a STL without pharyngeal resection, while 259 patients (64.1%) underwent a STL with partial pharyngectomy. Primary closure of the neopharynx without tissue transfer was performed in 173 patients (43.0%), while in the remainder of patients, a variety of healthy tissue transfer was used with the underlying idea to minimize postoperative complications. Most commonly used tissue transfer was onlay PM muscle flap in 131 patients (32.6%) and inset myocutaneous PM flap in 94 patients (23.4%). The decision to use tissue transfer for reconstruction depended on the pharyngeal defect after tumor ablation and was also influenced by institutional practice/surgeon's preference. Twenty percent of patients (*n* = 48) underwent a total thyroidectomy, while ~64% (*n* = 153) underwent a partial thyroidectomy. Nearly all patients (95.5%) received definitive STL as a treatment modality without adjuvant therapies. Eighteen patients (4.5%) underwent adjuvant treatment, most often re-irradiation, after STL.

**Table 5:** Surgery/procedural characteristics.

**Surgery/procedural characteristics**	***n***	**%**
**Type of salvage operation (*****n*** **=** **404)**		
Total laryngectomy	145	35.89
Total laryngectomy with partial pharyngectomy	259	64.11
**Reconstruction type (*****n*** **=** **402)**		
None	173	43.03
PM flap muscle onlay	131	32.59
Myocutaneous PM flap	94	23.38
Radial forearm free flap	3	0.75
Other	1	0.25
**Extent of neck dissection (*****n*** **=** **400)**		
None	121	30.25
Ipsilateral	136	34.00
Bilateral	143	35.75
**Tracheo-esophageal puncture (*****n*** **=** **368)**		
None	32	8.70
Primary	320	86.96
Secondary	16	4.35
**Adjuvant treatment STL (*****n*** **=** **398)**		
No adjuvant treatment	380	95.48
Adjuvant RT (reirradation)	14	3.52
Adjuvant CRT (including reirradiation)	2	0.50
Other	2	0.50

Mean and median follow-up were 8.3 and 7.9 years, respectively (Kaplan–Meier estimate of potential follow-up). Postoperative complications are illustrated in Table 6. The mean duration of hospital stay was 21 days (SD 16.3 days, range 8–162 days). Thirty-six patients (15.1%) had to be readmitted to the hospital within 1 month (“early readmission”), most frequently as a result of a late PCF development (*n* = 26). The mean duration of hospital stay after early readmission was 11 days (SD 9.5 days, range 2–44 days). Early postoperative complications (clinical PCF, wound infection, and hemorrhage) occurred in 134 patients (34.2%): PCF was the most common complication (*n* = 100, 25.5%) and was conservatively treated in the majority of patients affected (*n* = 87, 87%). Postoperative hemorrhage and wound infections were reported in 21 patients (5.4%) and 63 patients (16.2%), respectively. One patient (0.3%) died within 30 days after STL due to the consequences of a myocardial infarction. The global rate of permanent hypoparathyroidism necessitating long-term calcium substitution and administration of alfacalcidol (1-Alpha® Leo) was 5.4% (20/372) or 35.1% (20/57) in the subgroup of patients who underwent a total thyroidectomy during STL. Surgical revision of the tracheostoma, indicated for narrowed tracheostomas leading to hygienic problems or breathing discomfort, proved necessary in 1.9% (7/372) of cases during follow-up.

**Table 6:** Complications and functional outcomes.

**Postoperative complications**	***n***	**%**	**Functional results**	***n***	**%**
Hospital stay STL (days)			Days to resume peroral feeding		
*N*	394		N	328	
Mean	21.34		Mean	19.88	
Std	16.272		Std	25.795	
Median	16.00		Median	12.00	
IQR	(14.00–22.00)		IQR	(10.00–19.00)	
Range	(8.00–162.00)		Range	(4.00–253.00)	
Early readmission (*n* = 238)			Achievement total peroral intake (*n* = 362)		
No	202	84.87	No	21	5.80
Yes	36	15.13	Yes	341	94.20
Yes, late fistula related	26	10.92	Achievement functional speech (*n* = 352)		
Yes, stoma related	1	0.42	No	47	13.35
Yes, feeding related	1	0.42	Yes	305	86.65
Yes, infection related Yes, general health	44	1.681.68	Type speech (*n* = 341) Esophagus speech	11	3.23
Duration early readmission (days)			TEP speech	321	94.13
*N*	36		Electrolarynx speech	9	2.64
Mean	11.08		Permanent tube feeding (*n* = 363)		
Std	9.464		No	343	94.49
Median	8.50		Yes	20	5.51
IQR	(4.00–15.00)		Dysphagia (*n* = 351)		
Range	(2.00–44.00)		No	241	68.66
Salivary leakage^*^ (*n* = 234)			Yes	110	31.34
No	168	71.79	Dysphagia treatment (*n* = 110)		
Yes	66	28.21	No treatment	82	74.55
Early complication (PCF, hemorrhage, wound infection) (*n* = 392)			Endoscopic dilatation Neopharyngeal reconstruction	26 2	23.64 1.82
No	258	65.82			
Yes	134	34.18			
Pharyngocutaneous fistula (*n* = 392)					
No	292	74.49			
Yes	100	25.51			
Fistula treatment (*n* = 100)					
Conservative therapy	87	87.00			
Surgical closure with flap	12	12.00			
Surgical revision	1	1.00			
Hemorrhage (*n* = 390)					
No	369	94.62			
Yes	21	5.38			
Wound infection (*n* = 390)					
No	327	83.85			
Yes	63	16.15			

* *Salivary leakage first postoperative gastrografin esophagram*.

### Relationship Between Esophagram Results and PCF Formation

Postoperative esophagram results were collected from 234 UZ Leuven patients with the goal to examine the relationship between esophagram findings and the presence of or evolution toward clinical PCF formation. One hundred sixty-eight patients (71.8%) showed no salivary leakage on their first postoperative esophagram, while 66 patients (28.2%) did show a neopharyngeal dehiscence and beginning fistula development. Of these 66 patients, 34 (51.5%) also developed a clinical PCF during the later postoperative course, while 32 (48.5%) did not. In the group of 168 patients with a satisfactory first postoperative esophagram, 15.5% (*n* = 26) developed a delayed clinical PCF after start of oral intake and were eventually readmitted to the hospital. As such, delayed PCF formation was an important reason for hospital readmission. Comparing this subgroup of patients with the subgroup of patients with a satisfactory fluoroscopy who did not develop a clinical PCF, the former group of patients underwent significantly more chemoradiation (when compared to RT alone) as treatment modality for the first tumor (*p* = 0.026, Fisher's exact test). No differences between both groups were identified in relation to tumor stage, cTNM classification, pTNM classification, type of salvage operation (TL vs. TL with partial pharyngectomy), or reconstruction type.

A pharyngeal dehiscence observed on esophageal fluoroscopy without presence of a (clinical) PCF can be considered a “subclinical PCF.” When both clinical and subclinical fistulas (data of the latter were only available for UZ Leuven patients) were taken into account, the “overall PCF” incidence in UZ Leuven was 40.3% (96/238).

### Functional Outcomes

Functional outcomes are illustrated in Table 6. Eventual complete oral intake was achieved in 341 patients (94.2%). The mean and median time intervals between STL and resumption of peroral feeding were 19.9 and 12.0 days, respectively (SD 25.8 days, range 4–253 days). Twenty patients (5.5%) remained long-term tube feeding-dependent (G-tube or jejunostomy). Postoperative dysphagia, as subjectively reported by the patients during follow-up, proved a common complaint and occurred in 110 patients (31.3%), but did not require any treatment in most cases (*n* = 82, 74.6%) because of the mild severity. In more serious cases of dysphagia with an impact on peroral intake, surgical management of neopharyngeal stenosis by endoscopic dilations (Savary or balloon dilations, *n* = 26 or 7.4%) or neopharyngeal reconstruction (*n* = 2 or 0.6%) proved necessary. Regarding speech rehabilitation, 86.7% of patients (*n* = 305) achieved a functional speech, which was most frequently achieved by TEP speech (94.1%). In total, 90.7% of TEP patients achieved functional speech. Primary TEP (during STL) was performed in 320 patients (86.7%), of whom 90.3% achieved functional speech, while secondary puncture was performed in only 16 cases (4.4%), of whom 100% achieved functional speech.

### Factors Predictive for Early Postoperative Complications and PCF Formation

Treating center, preoperative Hb, postoperative Hb, and treatment modality of the initial/primary tumor were significantly associated with overall postoperative complication rates (including PCF, postoperative bleeding, and infection) upon univariable analysis, with lower Hb levels (<12.5 g/dl) and chemoradiation (when compared to RT alone) increasing the probability for a complicated postoperative course. However, only preoperative Hb remained a significant prognostic factor in multivariable analysis (OR = 0.579, *p* = 0.0241). Minor but significant differences were seen in overall complication rate between participating centers (ranging from 11.1% to 40.6%, *p* = 0.021 on Fisher's exact test) and treatment center was identified as a prognostic factor for complication rate (*p* = 0.0463) on univariable analysis. However, after correcting for potential confounders (cT, cN, pT, pN, tumor stage, type of salvage operation, and reconstruction type), the treatment center could not be confirmed as a significant risk factor for overall complication rate (*p* = 0.19).

No significant prognosticators for clinical PCF formation specifically were identified, neither on univariable nor on multivariable analysis. Clinical PCF incidence proved comparable among centers (ranging from 11.11 to 37.50%, *p* = 0.142 on Fisher's exact test). Table 1 lists the results of univariable analysis. No confounding factors were identified. However, when performing univariable analysis in the subgroup of patients from 1 center (UZ Leuven) with clinical and subclinical PCFs taken together (“overall PCF”), higher preoperative hemoglobin (>12.5 g/dl) (OR = 0.55, *p* = 0.03) and reconstruction with an onlay PM flap when compared to no flap (OR = 0.50, *p* = 0.04) or to myocutaneous PM flap (OR = 0.31, *p* < 0.01) were associated with a lower probability of (clinical or subclinical) fistula formation, while chemoradiation as a treatment of the first tumor (when compared to RT; OR = 3.15, *p* < 0.01) was identified as a negative prognosticator for overall fistula formation. On multivariable analysis, the use of a PM muscle onlay was confirmed to have a protective effect in preventing overall fistula formation when compared to no PM flap (OR = 0.36, *p* < 0.01) or inset myocutaneous PM flap (OR = 0.29, *p* < 0.01), while the negative prognostic effect of prior radiochemotherapy (OR = 3.77, *p* ≤ 0.01) could also be confirmed.

### Factors Predictive for Functional Outcomes

#### Non-achievement of Peroral Intake

Tables 7, 8 show the uni- and multivariable analysis of variables potentially related to non-achievement of total peroral intake. cN classification of the second tumor was identified as a possible confounder and this was corrected for in the analysis. Reconstruction type, treatment of the initial tumor, development of postoperative complications in general and PCF in particular and section margin status all proved significantly associated with non-achievement of total peroral intake on univariable analysis. The use of an onlay PM muscle flap or inset myocutaneous PM flap was significantly associated with a lower probability of achieving total peroral intake when compared to primary neopharyngeal closure without tissue transfer (*p* = 0.0057, and *p* = 0.0122, respectively). Section margin status proved to have a significant prognostic impact with non-achievement of total peroral intake being less likely when free margins could be obtained (*p* < 0.01). In the subgroup of patients with a residual or recurrent tumor, primary treatment by surgery and adjuvant RT proved a negative prognostic factor (*p* = 0.0249). Upon multivariable analysis, treatment of the initial tumor, PCF formation during the postoperative period, and free margin status remained significant prognosticators. Patients who postoperatively developed a PCF were less likely to eventually achieve total peroral intake (OR = 4.5, *p* = 0.0052), while patients with a free section margin status were more likely to achieve total peroral intake (OR = 0.179, *p* = 0.0132). Multivariable analysis applied to the subgroup with residual/recurrent tumors confirmed the negative impact of surgical treatment with adjuvant RT for the first tumor as compared to definitive RT (OR = 11.8, *p* = 0.0134). Definite CRT (compared to definitive RT) showed a trend toward worse outcome in the recurrent/residual group (OR=3.5, *p* = 0.0975) and in the total group (OR = 3.0, *p* = 0.0854). When applied to the subgroup with second primary tumors, no significant impact of treatment modality of the first tumor could be identified.

**Table 7:** Prognostic factors for non-achievement total peroral intake with correction for confounding (univariable).

**Variables**	**Univariable Prognostic factors**		
	**OR**	**95% CI**	***p-*value**
Type of salvage operation			
STL+partial pharyngectomy vs. STL	2.567	0.668–9.860	0.1698
Reconstruction type			
Global test			**0.0179**
PM flap muscle onlay vs. myocutaneous PM flap	1.051	0.370–2.983	0.9256
PM flap muscle onlay vs. none	7.577	1.803–31.851	**0.0057**
Myocutaneous PM flap vs. none	7.210	1.539–33.776	**0.0122**
Type of second tumor			
Global test			0.2222
Local recurrence vs. Residual tumor	0.442	0.132–1.478	0.1849
Local recurrence vs. Second primary	0.316	0.084–1.186	0.0877
Residual tumor vs. Second primary	0.716	0.243–2.107	0.5443
Treatment initial tumor (residual/recurrent)			
Surgery + adjuvant RT vs. definitive CRT	2.791	0.309–25.178	0.3603
Surgery + adjuvant RT vs. definitive RT	9.592	1.331–69.147	**0.0249**
Definitive CRT vs. definitive RT	3.436	0.883–13.377	0.0750
Treatment initial tumor (second primary)			
Surgery + adjuvant RT vs. definitive CRT	1.503	0.136–16.605	0.7398
Surgery + adjuvant RT vs. definitive RT	7.218	0.820–63.547	0.0749
Definitive CRT vs. definitive RT	4.804	0.508–45.391	0.1708
Radiotherapy initial tumor dose on larynx (Gray)			
+ 1 unit	1.080	0.954–1.224	0.2251
Tracheo-esophageal puncture			
Secondary/none vs. Primary	0.261	0.052–1.316	0.1036
Adjuvant treatment STL			
Yes (any type) vs. No	0.974	0.116–8.163	0.9809
Complication (any type)			
Yes (any type) vs. No	2.482	1.003–6.141	**0.0492**
Section Margins			
Free (>5 mm) vs. positive	0.119	0.037–0.384	**0.0004**
Pharyngocutaneous Fistula STL			
Yes (any type) vs. No	3.398	1.361–8.485	**0.0088**

*Binary variables/pairwise tests: OR > (<) 1: higher (lower) risk for first category. Reconstruction type: radial forearm free flap (n = 3) and other (n = 1) excluded. Bold values are statistically significant (p < 0.05)*.

**Table 8:** Prognostic factors for non-achievement total peroral intake (multivariable analysis).

**Variables**	**Multivariable Prognostic factors all tumors**			**Multivariable Prognostic factors residual/recurrent tumors**		
	**OR**	**95% CI**	***p-*value**	**OR**	**95% CI**	***p-*value**
Treatment initial tumor						
Global test			**0.0038**			0.0712
Surgery + adjuvant RT vs. definitive CRT	4.050	0.916–17.910	0.0651	3.365	0.423–26.741	0.2513
Surgery + adjuvant RT vs. definitive RT	12.159	3.159–46.798	**0.0003**	11.845	1.668–84.109	**0.0134**
Definitive CRT vs. definitive RT	3.002	0.858–10.502	0.0854	3.520	0.794–15.600	0.0975
Section margins						
Free (>5 mm) vs. positive	0.179	0.046–0.698	**0.0132**	0.396	0.062–2.541	0.3284
Pharyngocutaneous Fistula STL						
Yes (any type) vs. No	4.511	1.567–12.984	**0.0052**	5.676	1.532–21.031	**0.0094**

*Binary variables/pairwise tests: OR > (<) 1: higher (lower) risk for first category. Reconstruction type: radial forearm free flap (n = 3) and other (n = 1) excluded. Bold values are statistically significant (p < 0.05)*.

#### Permanent Tube Feeding

Table 9 shows the uni- and multivariable analysis of variables potentially related to postoperative permanent tube feeding. cN classification of the second tumor was again identified as a possible confounder; this was corrected for in analysis. Patients who experienced postoperative complications in general and PCF formation in particular were significantly more likely to stay tube feeding-dependent upon univariable analysis (OR = 3.5, *p* = 0.011 and OR = 3.7, *p* = 0.0062, respectively). Patients with free section margins were less likely to remain G-tube dependent (OR = 0.3, *p* = 0.044). Upon multivariable analysis, PCF and higher cN classification of the second tumor were identified as independent prognostic factors for long-term tube feeding dependency (OR = 3.7, *p* = 0.0062 and OR = 1.4, *p* = 0.0198, respectively).

**Table 9:** Prognostic factors for permanent tube feeding with correction for confounding.

**Variables**	**Univariable Prognostic factors**			**Multivariable Prognostic factors**		
	**OR**	**95% CI**	***p-*value**	**OR**	**95% CI**	***p*-value**
Type of salvage operation						
STL+partial pharyngectomy vs. STL	0.777	0.263–2.300	0.6488		-	
Reconstruction type						
PM flap muscle onlay vs. myocutaneous PM flap	1.183	0.362–3.864	0.7803		-	
PM flap muscle onlay vs. none	2.122	0.660–6.829	0.2069		-	
Myocutaneous PM flap vs. none	1.793	0.474–6.792	0.3899		-	
Type of second tumor						
Local recurrence vs. Residual tumor	0.756	0.232–2.462	0.6428		-	
Local recurrence vs. Second primary	0.404	0.115–1.416	0.1567		-	
Residual tumor vs. Second primary	0.534	0.172–1.660	0.2782		-	
Treatment initial tumor (residual/recurrent)^*^						
Surgery + adjuvant RT vs. definitive CRT	0.643	0.039–10.587	0.7577		-	
Surgery + adjuvant RT vs. definitive RT	2.369	0.187–29.954	0.5052		-	
Definitive CRT vs. definitive RT	3.682	0.918–14.770	0.0659		-	
Treatment initial tumor (second primary)^*^						
definitive CRT vs. definitive RT	3.631	0.442–29.854	0.2303		-	
Radiotherapy initial tumor dose on larynx (Gray)						
+1 unit	1.053	0.935–1.187	0.3908		-	
Tracheo-oesofageal puncture^*^						
Primary vs. secondary	0.625	0.075–5.232	0.6646		-	
Adjuvant treatment STL						
Yes (any type) vs. No	2.438	0.483–12.305	0.2805		-	
Complication (any type)						
Yes vs. No	3.477	1.330– 9.088	**0.0110**		-	
Section margins						
Free (>5 mm) vs. positive	0.279	0.080–0.966	**0.0440**		-	
Pharyngocutaneous Fistula STL						
Yes vs. No	3.739	1.454–9.615	**0.0062**	3.739	1.454–9.615	**0.0062**
Clinical nodal stage second tumor						
+1 level		-		1.371	1.051–1.788	**0.0198**

Binary variables/pairwise tests: OR > (<) 1: higher (lower) risk for first category. Reconstruction type: radial forearm free flap (n = 3) and other (n = 1) excluded.

* Not all pairwise comparisons were estimable due to small groups/zero events.

*Bold values are statistically significant (p < 0.05)*.

#### Post-operative Dysphagia

Table 10 depicts the uni- and multivariable analysis of variables potentially related to postoperative dysphagia. No confounders could be identified. Upon univariable analysis, development of postoperative complications and PCF, TL with partial pharyngectomy (compared to TL alone) and reconstruction with an onlay PM muscle flap (when compared to inset myocutaneous PM flap and to no tissue transfer) proved significant risk factors for postoperative dysphagia. The prognostic effect of the reconstruction modality was re-assessed in the subgroup of patients undergoing a TL without partial pharyngectomy and the negative impact of a muscle onlay PM flap on dysphagia, when compared to no tissue transfer, was confirmed in this subgroup (OR = 3.9, *p* = 0.0097). However, when the use of an inset myocutaneous PM flap was compared to the onlay muscle PM in the TL without partial pharyngectomy subgroup, the onlay PM flap did lose its negative prognostic effect on postoperative dysphagia (OR = 3.7, *p* = 0.2519). In the subgroup of patients who underwent STL for residual or recurrent tumors, treatment of the initial tumor proved to be an additional prognostic factor. Patients who underwent surgery combined with adjuvant RT as treatment of the initial tumor were more likely to experience postoperative dysphagia compared to definitive RT.

**Table 10:** Prognostic factors for postoperative dysphagia, in the total patient group and in the salvage total laryngectomy without pharyngectomy [TL] subgroup.

**Variables**	**Univariable Prognostic factors**			**Multivariable Prognostic factors**		
	**OR**	**95% CI**	***p-*value**	**OR**	**95% CI**	***p*-value**
Type of salvage operation						
STL+partial pharyngectomy vs. STL	2.680	1.559–4.607	**0.0004**	2.661	1.469–4.819	**0.0012**
Reconstruction type						
PM flap muscle onlay vs. myocutaneous PM flap	2.061	1.130–3.760	**0.0183**	2.527	1.350–4.728	**0.0037**
PM flap muscle onlay vs. myocutaneous PM flap [TL]	3.684	0.396–34.291	0.2519		-	
PM flap muscle onlay vs. none	2.932	1.711–5.025	**<0.0001**	2.716	1.536–4.803	**0.0006**
PM flap muscle onlay vs. none [TL]	3.918	1.392–11.026	**0.0097**		-	
Myocutaneous PM flap vs. none	1.422	0.779–2.596	0.2512	1.075	0.564–2.048	0.8257
Myocutaneous PM flap vs. none [TL]	1.063	0.117–9.673	0.9564		-	
Type of second tumor						
Local recurrence vs. Residual tumor	0.906	0.545–1.507	0.7046		-	
Local recurrence vs. Second primary	0.813	0.441–1.499	0.5073		-	
Residual tumor vs. Second primary	0.897	0.488–1.651	0.7272		-	
Treatment initial tumor (residual/recurrent)						
Surgery + adjuvant RT vs. definitive CRT	3.176	0.700;- 14.421	0.1343		-	
Surgery + adjuvant RT vs. definitive RT	4.951	1.200–20.430	**0.0270**		-	
Surgery + adjuvant RT vs. surgery + adjuvant CRT	6.000	0.422–85.248	0.1857		-	
Definitive CRT vs. definitive RT	1.559	0.793–3.063	0.1980		-	
Definitive CRT vs. surgery + adjuvant CRT	1.889	0.181–19.670	0.5947		-	
Definitive RT vs. surgery + adjuvant CRT	1.212	0.124–11.880	0.8689		-	
Treatment initial tumor (second primary)^*^						
Surgery + adjuvant RT vs. definitive CRT	1.875	0.302–11.626	0.4995		-	
Surgery + adjuvant RT vs. definitive RT	2.833	0.666–12.059	0.1587		-	
definitive CRT vs. definitive RT	1.511	0.371–6.149	0.5642		-	
Radiotherapy initial tumor dose on larynx (Gray)						
+1 unit	1.025	0.982–1.070	0.2556		-	
Tracheo-oesofageal puncture^*^						
Primary vs. secondary	3.015	0.662–13.727	0.1537		-	
Adjuvant treatment STL						
Yes (any type) vs. No	0.280	0.063–1.248	0.0952		-	
Complication (any type)						
Yes vs. No	2.026	1.273–3.225	**0.0029**		-	
Section margins						
Free (>5 mm) vs. positive	0.952	0.416–2.181	0.9081		-	
Pharyngocutaneous Fistula STL						
Yes vs. No	2.179	1.328–3.575	**0.0021**	2.406	1.424–4.065	**0.0010**

Binary variables/pairwise tests: OR > (<) 1: higher (lower) risk for first category. Reconstruction type: radial forearm free flap (n = 3) and other (n = 1) excluded.

* Not all pairwise comparisons were estimable due to small groups/zero events.

*Bold values are statistically significant (p < 0.05)*.

Upon multivariable analysis, type of salvage operation, reconstruction type, and postoperative PCF remained significant. Patients who developed PCF postoperatively were 2.4 times more likely to experience postoperative dysphagia compared to those without PCF (*p* = 0.0010). Dysphagia was 2.7 times less likely in patients treated only with TL compared to those who also underwent a partial pharyngectomy (*p* = 0.0012). Reconstruction with a muscle onlay PM flap proved independently associated with more dysphagia when compared to inset myocutaneous PM flap (OR = 2.5, *p* = 0.0037) and to primary closure without tissue transfer (OR = 2.7, *p* = 0.0006). However, upon multivariable analysis of the “total laryngectomy alone subgroup,” the reconstruction type lost its prognostic effect.

#### Non-achievement of Functional Speech

Upon univariable analysis, the total dose of administered RT on the larynx during irradiation of the primary tumor was identified as a continuous variable, which was significantly associated with the non-achievement of functional speech during the postoperative course, with higher doses implying higher risk for non-achievement of functional speech. However, the administered dose of RT on the larynx lost its prognostic significance after correcting for pN classification as a confounder. Additionally, an effect of margin status on non-achievement of functional speech was observed, with free margins acting as a negative prognosticator for non-achievement of functional speech/positive prognosticator for functional speech (OR = 0.192, *p* < 0.01). Multivariable analysis confirmed this independent prognostic effect of section margin status (OR = 0.175, *p* < 0.01) and identified pT classification as an additional prognosticator with higher pT classifications related to a higher chance of not achieving functional speech (OR = 1.45, *p* < 0.01). Table 11 shows the univariable analysis of variables potentially related to non-achievement of functional speech, with correction for confounding factors.

**Table 11:** Prognostic factors for non-achievement functional speech with correction for confounding (univariable).

**Variables**	**Prognostic factors**		
	**OR**	**95% CI**	***p-*value**
Type of salvage operation			
STL+partial pharyngectomy vs. STL	0.839	0.431–1.635	0.6068
Reconstruction type			
PM flap muscle onlay vs. myocutaneous PM flap	1.378	0.579–3.281	0.4683
PM flap muscle onlay vs. none	1.175	0.567–2.432	0.6644
Myocutaneous PM flap vs. none	0.852	0.369–1.970	0.7085
Type of second tumor			
Local recurrence vs. Residual tumor	1.491	0.713–3.118	0.2891
Local recurrence vs. Second primary	1.414	0.578–3.460	0.4475
Residual tumor vs. Second primary	0.949	0.385–2.340	0.9092
Treatment initial tumor (residual/recurrent)			
Surgery + adjuvant RT vs. definitive CRT	1.140	0.115–11.334	0.9107
Surgery + adjuvant RT vs. definitive RT	1.332	0.153–11.578	0.7950
Definitive CRT vs. definitive RT	1.168	0.442–3.088	0.7544
Treatment initial tumor (second primary)			
Surgery + adjuvant RT vs. definitive CRT	0.789	0.079–7.929	0.8405
Surgery + adjuvant RT vs. definitive RT	2.267	0.337–15.226	0.3998
definitive CRT vs. definitive RT	2.872	0.430–19.181	0.2761
Radiotherapy initial tumor dose on larynx (Gray)			
+1 unit	1.067	0.990–1.149	0.0887
Adjuvant treatment STL			
Yes (any type) vs. No	1.787	0.488–6.542	0.3809
Complication (any type)			
Yes vs. No	1.176	0.614–2.252	0.6247
Section Margins			
Free (>5 mm) vs. positive	0.192	0.078–0.471	**0.0003**
Pharyngocutaneous Fistula STL			
Yes vs. No	1.311	0.655–2.624	0.4451

Continuous variables: OR > (<) 1: increased (decreased) risk with increasing variable level. Binary variables/pairwise tests: OR > (<) 1: higher (lower) risk for first category.

Reconstruction type: radial forearm free flap (n = 3) and other (n = 1) excluded.

*Bold values are statistically significant (p < 0.05)*.

### Prognostic Factors for Hospital Stay Duration

Multiple significant prognostic factors were identified for hospital stay duration upon univariable analysis and are listed in Table 12. Upon multivariable analysis, myocutaneous PM flap reconstruction was significantly associated with longer hospital stay. Patients who underwent myocutaneous PM flap reconstruction had a 30.5% increase in hospital stay compared to patients who had no flap reconstruction (relative effect or RE = 1.305, *p* ≤ 0.0001); the latter had a 21.1% increase compared to PM flap muscle onlay patients (RE = 0.789, *p* ≤ 0.0001). Patients without TEP stayed longer in the hospital compared to those who underwent a primary or secondary TEP (15.9 and 36.7% increase respectively). This finding most likely represents a selection bias to the fact that the decision to refrain from TEP is preferentially made in patients with significant comorbidities, and/or poor tissue quality, making them more susceptible to postoperative complications and longer hospital stay. Postoperative complications (any) and clinical PCF prolonged hospitalization by 41.5% (RE = 1.415; *p* ≤ 0.0001) and 24.6% (RE = 1.246; *p* = 0.0030), respectively.

**Table 12:** Prognostic factors for hospital stay.

**Variables**	**Univariable Prognostic factors**			**Multivariable Prognostic factors**		
	**RE**	**95% CI**	***p*-value**	**RE**	**95% CI**	***p-*value**
Type of second tumor						
Local recurrence vs. Residual tumor	0.897	0.811–0.992	**0.0340**		-	
Local recurrence vs. Second primary	0.843	0.744–0.956	**0.0078**		-	
Residual tumor vs. Second primary	0.940	0.829–1.067	0.3390		-	
Initial location second tumor						
Combination vs. glottic	1.041	0.798–1.357	0.7694		-	
Combination vs. hypopharynx	0.697	0.507–0.960	**0.0269**		-	
Combination vs. subglottic	1.051	0.764–1.447	0.7596		-	
Combination vs. supraglottic	1.017	0.777–1.332	0.9013		-	
Combination vs. transglottic	1.041	0.781–1.388	0.7826		-	
Glottic vs. hypopharynx	0.670	0.548–0.820	**<0.0001**		-	
Glottic vs. subglottic	1.010	0.826–1.236	0.9215		-	
Glottic vs. supraglottic	0.978	0.878–1.088	0.6783		-	
Glottic vs. transglottic	1.001	0.865–1.157	0.9925		-	
Hypopharynx vs. subglottic	1.508	1.153–1.972	**0.0027**		-	
Hypopharynx vs. supraglottic	1.459	1.186–1.795	**0.0003**		-	
Hypopharynx vs. transglottic	1.493	1.188–1.878	**0.0006**		-	
Subglottic vs. supraglottic	0.968	0.787–1.190	0.7563		-	
Subglottic vs. transglottic	0.991	0.788–1.246	0.9357		-	
Supraglottic vs. transglottic	1.024	0.878–1.193	0.7647		-	
Pathological tumor classification second tumor						
+1 level	0.992	0.960–1.026	0.6508		-	
Pathological nodal classification second tumor						
+1 level	1.032	0.999–1.066	0.0575		-	
Overall tumor stage second tumor						
+1 level	1.063	1.016–1.112	**0.0082**		-	
Preoperative active smoking						
Yes vs. No	1.041	0.933–1.161	0.4695		-	
Preoperative tracheotomy						
Yes vs. No	1.128	0.959–1.327	0.1444		-	
Extent of neck dissection						
Bilateral vs. Ipsilateral	0.996	0.890–1.115	0.9461		-	
Bilateral vs. None	1.034	0.919–1.163	0.5822		-	
Ipsilateral vs. None	1.038	0.921–1.169	0.5427		-	
Section margins second tumor						
Close margin (<5 mm) vs. Free margins (>5 mm)	1.150	0.996–1.328	0.0569		-	
Close margin (<5 mm) vs. Positive margin	1.168	0.943–1.446	0.1551		-	
Free margins (>5 mm) vs. Positive margin	1.015	0.854–1.208	0.8640		-	
Preoperative Hb						
≥12.5 g/dl vs. <12.5 g/dl	0.914	0.825–1.012	0.0830		-	
Postoperative Hb						
≥12.5 g/dl vs. <12.5 g/dl	0.797	0.711–0.894	**0.0001**		-	
Preoperative albumin						
≥3.5 g/dl vs. <3.5 g/dl	0.953	0.787–1.153	0.6177		-	
Postoperative albumin						
≥3.5 g/dl vs. <3.5 g/dl	0.901	0.743–1.093	0.2901		-	
Type of salvage operation						
STL+partial pharyngectomy vs. STL	1.023	0.926–1.129	0.6542	0.916	0.839–0.999	**0.0475**
Reconstruction type						
PM flap muscle onlay vs. myocutaneous PM flap	0.808	0.713–0.915	**0.0008**	0.789	0.713–0.873	**<0.0001**
PM flap muscle onlay vs. none	1.052	0.944–1.171	0.3593	1.030	0.939–1.130	0.5277
Myocutaneous PM flap vs. none	1.301	1.156–1.464	**<0.0001**	1.305	1.178–1.446	**<0.0001**
Treatment initial tumor (residual/recurrent)						
Surgery + adjuvant RT vs. definitive CRT	1.017	0.740–1.399	0.9166		-	
Surgery + adjuvant RT vs. definitive RT	1.095	0.814–1.473	0.5507		-	
Surgery + adjuvant RT vs. surgery + adjuvant CRT	0.995	0.611–1.619	0.9823		-	
Definitive CRT vs. definitive RT	1.076	0.935–1.238	0.3064		-	
Definitive CRT vs. surgery + adjuvant CRT	0.978	0.648–1.476	0.9145		-	
Definitive RT vs. surgery + adjuvant CRT	0.909	0.612–1.349	0.6343		-	
Treatment initial tumor (second primary)						
Surgery + adjuvant RT vs. definitive CRT	0.788	0.511–1.217	0.2825		-	
Surgery + adjuvant RT vs. definitive RT	1.079	0.772–1.507	0.6578		-	
Surgery + adjuvant RT vs. surgery + adjuvant CRT	1.562	0.564–4.324	0.3907		-	
Definitive CRT vs. definitive RT	1.368	0.979–1.912	0.0662		-	
Definitive CRT vs. surgery + adjuvant CRT	1.982	0.716–5.486	0.1880		-	
Definitive RT vs. surgery + adjuvant CRT	1.448	0.544–3.858	0.4589		-	
Radiotherapy initial tumor dose on larynx (Gray)						
+1 unit	0.992	0.984–1.000	**0.0491**	0.990	0.984–0.996	**0.0021**
Tracheo-oesofageal puncture						
None vs. primary	1.208	1.009–1.446	**0.0394**	1.159	1.004–1.339	**0.0441**
None vs. secondary	1.256	0.939–1.681	0.1247	1.367	1.081–1.727	**0.0089**
Primary vs. secondary	1.040	0.817–1.324	0.7499	1.179	0.973–1.428	0.0936
Complication (any type)						
Yes vs. No	1.673	1.535–1.825	**<0.0001**	1.415	1.238–1.616	**<0.0001**
Pharyngocutaneous Fistula STL						
Yes vs. No	1.701	1.545–1.874	**<0.0001**	1.246	1.078–1.442	**0.0030**

Continuous variables: RE > (<) 1: increased (decreased) hospital stay with increasing variable level. Binary variables/pairwise tests: RE > (<) 1: longer (shorter) hospital stay for first category.

e.g., RE = 1.10 means 10% increase.

Reconstruction type: radial forearm free flap (n = 3) and other (n = 1) excluded.

*Bold values are statistically significant (p < 0.05)*.

## Discussion

Surgical complications prolong the length of hospital stay, negatively impact healthcare and costs, and delay initiation of peroral feeding and rehabilitation, consequently worsening global outcomes. Complication rate is higher in salvage patients, due to the prior radiation's effect on the tissue's microvasculature and healing capacities (11, 13). We report a complication rate of 34.2% after STL, including clinical PCF formation, postoperative bleeding, and infection. Overall complication rates are difficult to compare with previous studies because of heterogeneity of the population studied and the specification of complication types included in the analysis. Similar to our definition, several studies defined postoperative bleeding as wound hematoma requiring surgical revision (25–27). Furuta et al. reported postoperative hemorrhages in 5.8% (*n* = 5) of their STL patients, which is comparable to our rate of 5.4% (*n* = 21) (27). Individual studies have reported wound infection rates between 0.8 and 45% (28, 29). In our series, local wound infection occurred in 16.15% (*n* = 63) of the patients. However, local wound infection in previous studies was not clearly defined and patient population was not uniform regarding applied surgical techniques and postoperative care. With a rate of 25.5%, clinical PCF formation was the most frequent complication after STL in our series, which is similar compared to the reported PCF pooled incidence in a recent systematic review (28.9%) (14). However, when both clinical and subclinical fistulas were taken into account upon subset analysis, the “overall PCF” incidence raised to 40.3%. Given the high rates of complications and their association with significant morbidity, it is highly relevant to identify potential risk factors for overall complications and clinical PCF formation in particular. In univariable analysis, low preoperative hemoglobin (Hb), low postoperative Hb, and treatment of the primary tumor by CRT (compared to RT) were significantly associated with higher overall postoperative complication rates. In previous studies, prior CRT proved, parallel to our findings, to be related with higher complication rates (2, 10, 13, 30–33). Possible explanations for this finding include the more extended toxic effect on tissues by CRT compared to RT alone and the higher probability of impaired nutritional and immune status in patients receiving additional chemotherapy. Finally, CRT is indicated in patients diagnosed with higher staged tumors, resulting in an important selection bias of the patients treated with CRT (1, 7, 34). However, in multivariable analysis, only preoperative hemoglobin (≥12.5 g/dl) proved to be a significant positive prognosticator related to a decreased overall postoperative complication rate.

No significant risk factors for clinical PCF formation specifically could be identified, on univariable or on multivariable analysis. However, univariable analysis did show a strong trend toward significance with a negative effect of low postoperative Hb value (<12.5 g/dl) (*p* = 0.055), which is not surprising given the fact that postoperative anemia is known to be associated with worse healing capacities (35). Primary TEP could not be identified as a risk factor for PCF formation in this study, confirming once more the safety of primary TEP in patients undergoing STL (36). Quite surprising, vascularized flap tissue reconstruction was not confirmed to be a significant factor preventing PCF formation, which is in line with various previously published results (16, 31, 37–42). In a study by Benson et al., PCF rates even increased when PM coverage was used (43). However, an inherent selection bias must be taken into account: more advanced carcinomas are more likely to undergo flap reconstruction using PM flaps or free tissue transfer. Nevertheless, tumor stage was not identified as a confounding factor in our analysis. Other publications however have shown a protective effect. Two recent systematic reviews examined the impact of vascularized tissue transfers on reducing fistula following STL (12, 44). Patel et al. reported a study population of 359 STL patients from eight institutions and observed decreased fistula rates when either free tissue transfer (25% PCF rate) or PM overlay (15% PCF rate) was used, as compared to primary closure (34% PCF rate) (44). Paleri et al. included seven studies (591 patients) in a systematic review and found similar results to Patel et al.: they found that fistula formation was reduced by roughly one-third in patients who underwent vascularized flap reconstruction (22.2%) compared to those who underwent primary closure (31.2%) (12). The principle behind vascularized flap reconstruction is that it will improve wound healing and prevent fistula formation by using non-irradiated, well-vascularized tissue. These contradicting findings described in the literature result in unclarities as to the ideal method of reconstruction during STL in order to reduce PCF incidence. Although no significant risk factors for clinical PCF formation could be identified in the total population, when repeating univariable analysis in a subgroup of patients from one center with clinical and subclinical PCFs taken together (“overall PCF”), higher preoperative hemoglobin (>12.5 g/dl) and onlay PM flap (when compared to no flap or myocutaneous PM flap) were associated with less fistula formation, while chemoradiation as a treatment of the first tumor (when compared to RT), was identified as a negative prognosticator for overall fistula formation. On multivariable analysis, the use of a PM muscle onlay was confirmed to have a protective effect while the negative prognostic effect of prior radiochemotherapy could also be confirmed. The inset myocutaneous PM flap's inferiority in preventing overall fistula formation can potentially be explained by the long skin-pharyngeal mucosa suture line when compared to the shorter classic T-closure of the neopharynx in case of onlay PM flap reconstruction.

We also examined the relation between findings on the first postoperative esophagram/esophageal fluoroscopy and evolution to clinical PCF formation. In our series, salivary leakage on the first postoperative esophagram was apparent in 28.2% of patients with approximately half of them eventually developing a clinical PCF during the later postoperative course. In patients with a satisfactory first postoperative esophagram, 15% developed a delayed clinical PCF after start of oral intake, which was an important reason for hospital readmission. Comparing this subgroup of patients with the subgroup of patients with a satisfactory fluoroscopy who did not develop a clinical PCF, the former group of patients underwent significantly more chemoradiation (when compared to RT alone) as treatment modality for the first tumor, suggesting these patients to be at a higher risk for late PCF development. As a conclusion, a normal postoperative esophagram did not guarantee an uncomplicated further evolution and radiographically evident fistulas did not evolve to clinical apparent fistulas in all cases. The latter finding can probably be explained by the policy of postponing peroral feeding after a positive esophagram, potentially preventing evolution to clinical fistula formation. However, Krouse et al. found esophagram results to be predictive of, but not preventive for, PCF formation in patients following (non-salvage) TL (45). The sensitivity of the esophagram in their study was found to be 55%, with a specificity of 80%. In another study by White et al., the sensitivity in salvage patients was only 14%, with a specificity of 91% (46). The exact reason for the high rate of patients with a satisfactory esophageal fluoroscopy eventually developing a clinical PCF is unclear and could be related to suboptimal sensitivity of the esophageal fluoroscopy using the aqueous low osmolar contrast agent iopromide (Ultravist®), potentially failing to detect small neopharyngeal dehiscences, which can evolve to a clinical PCF when peroral feeding is initiated. Although iopromide has fewer side effects while diagnosing a major leak (e.g., in case of fistula to the tracheostoma or trachea), this contrast medium is lighter and penetrates less as compared to barium. It has been shown that barium has superior physical properties of mucosal coating and radiographic density and is better in detecting esophageal perforations when compared to aqueous contrast media (47). As a consequence, small defects in the neopharynx might be overlooked during the Ultravist® swallow and an additional fluoroscopy using barium in these patients with a normal Ultravist® fluoroscopy could potentially lead to a higher detection rate of subclinical fistulas, preventing premature restart of oral intake, and as such, potentially preventing evolution to a clinical PCF. As we observed a higher proportion of patients who underwent chemoradiation in the group of patients with normal fluoroscopy eventually developing a clinical PCF, an additional explanation for this phenomenon could be related to delayed wound healing and consequently delayed fistula formation when the watertight neopharyngeal sutures are absorbing. As a consequence, we suggest, even after a favorable esophageal fluoroscopy, a slow and gradual progression in consistencies taken by mouth, especially in those patients who underwent CRT in the past. We advise our STL patients to drink clear fluids the first day after the satisfactory fluoroscopy, followed by liquid and semi-liquid foods for 3 weeks. Moreover, a first postoperative checkup is scheduled 1 week after hospital discharge, in order to detect symptoms and signs of late PCF formation. Progressive TEP speech rehabilitation, avoiding high neopharyngeal pressure buildup, is advised during the first postoperative weeks.

Functional outcomes after STL proved satisfactory in our series. Most patients in our study eventually achieved total peroral intake (94.2%; *n* = 341), which is a higher rate than reported in a study by van der Putten et al. (84%; *n* = 94) (28). However, 31% (*n* = 110) of our patients reported subjective dysphagia in the postoperative period, with the majority being of a mild nature and being managed by conservative measures (74.6% of cases) such as adjustment of food consistency. A systematic review by Hasan et al. reported an average dysphagia rate of 18.6% (incidence 2.9–30.2%), though the authors highlighted the problem of heterogeneity in dysphagia's definition among the included studies (14). In our cohort, single or serial dilations of neopharyngeal strictures proved necessary in 23.6% of patients complaining of dysphagia, or in 7.4% of the total STL population. In a recent paper by Petersen et al., assessing dilation incidence after TL, a dilation rate of 20.9% was observed in the STL group (*n* = 211). When taking all patients into account who underwent (C)RT before TL, a dilation rate of 26.8% was reported. On multivariable analysis, the salvage setting could not be identified as an independent risk factor for developing strictures necessitating dilation, but previous (C)RT was clearly associated with a higher dilation rate (HR = 6.13, *p* = 0.003) (48). However, given the discrepancy between patient populations who underwent previous (C)RT and those who underwent a STL, it is not quite clear how STL was defined.

In our series, the occurrence of complications and PCF formation during the postoperative course were identified as significant risk factors for the non-achievement of peroral intake, permanent tube feeding, and dysphagia in univariable analysis. In multivariable analysis, clinical PCF formation remained an independent risk factor for these swallowing outcomes. This confirms the findings by Chepeha et al. who observed that patients who acquired a postoperative fistula requiring reoperation were ~3.3 times more likely to have worse swallowing outcomes. However, PCF that healed without surgical treatment was not identified as a significant risk factor (49). Additionally, the reconstruction type significantly impacted upon various swallowing outcomes in univariable analysis. Patients undergoing reconstruction with muscle onlay or myocutaneous PM flaps were significantly less likely to achieve total peroral intake. However, reconstruction type did not prove to be a significant and independent prognosticator in multivariable analysis. The use of vascularized tissue augmentation has been linked to worse nutritional outcomes in previous studies, but the impact has so far been understudied (38, 49, 50). Furthermore, PM muscle onlay reconstruction was independently associated with more dysphagia when compared to no tissue transfer or neopharyngeal augmentation with an inset myocutaneous PM flap. It has been suggested that the bulkiness of the flap tissue might change swallowing dynamics of the neopharynx, causing swallowing difficulties (50). Additionally, these results could be subject to treatment selection bias by surgeons who are more likely to use flap reconstruction in more advanced disease necessitating more advanced resections, as well as in more heavily radiation therapy-damaged tissue. However, tumor stage was not identified as a confounding factor in our analysis for dysphagia outcomes, and both reconstruction type and type of salvage operation (TL vs. TL with partial pharyngectomy) were independent prognosticators on multivariable analysis, with dysphagia being 2.66 times more likely in patients treated with TL and partial pharyngectomy compared to those who only underwent a TL without pharyngectomy. A recent study by Worley et al. also observed a progressive increase in stricture rate and resulting dysphagia with increasing extent of pharyngectomy (51). In the total patient group (both TL patients and patients with TL and partial pharyngectomy), reconstruction with a muscle onlay PM flap proved independently associated with more dysphagia when compared to inset myocutaneous PM flap and to primary closure without tissue transfer. Given the fact that during salvage surgery obtaining free margins is of the utmost importance, a partial pharyngectomy is often necessary (64.1% of patients in our population), even in cases of laryngeal recurrences or laryngeal residual tumors. Due to our findings of higher dysphagia probability in patients with muscle onlay PM flaps, regardless its protective effect for overall (clinical and subclinical) fistula formation, we would suggest to prefer a myocutaneous inset PM flap over an onlay PM flap when free tissue transfer is considered desirable.

Treatment of the initial tumor also proved to have a significant effect on swallowing outcomes: patients with a residual/recurrent tumor who underwent surgery combined with adjuvant RT for their first tumor were less likely to achieve total peroral intake when compared to RT alone (OR = 11.8).

A high rate of functional speech was observed in this study (86,5%; *n* = 305), which is comparable to rates reported in other publications on STL populations (28, 42). Interestingly, reconstruction type did not impact voice outcomes in this study, which is in contrast to findings by Chepeha et al. (49). The authors did not observe statistically significant differences in speech outcomes between primary closure and vascularized tissue augmentation without muscle, but did report worse speech outcomes in patients with muscle reconstruction. Moreover, our univariable analysis displayed that patients receiving a higher dose of RT on the larynx during irradiation of the primary tumor were less likely to achieve speech rehabilitation and functional speech, but this effect was lost after correcting for confounding variables. The possible effect of radiotherapeutic dose distribution during primary tumor irradiation on speech outcomes after STL has not been studied so far. A prospective trial is needed to confirm or reject this potential relation. Multivariable analysis identified pT classification of the second tumor as an independent prognostic factor with the higher the pT classification, the lower the chance of achieving functional speech. Of interest, a prognostic effect of margin status on non-achievement of functional speech as well as on non-achievement of total peroral intake was observed, with free margins acting as a positive prognosticator for achievement of total peroral intake and functional speech upon multivariable analysis. This finding can potentially be explained by the higher probability of administering adjuvant re-irradiation to patients with positive section margins, leading to worse functional outcomes.

There are some limitations in the present study. As the current study analyzed data retrospectively, selection bias is inherent to the design. Moreover, we collected data from four different reference hospitals, and although these hospitals have a similar general approach and philosophy, there was no standardization in decision-making or in therapy (e.g., choice of reconstruction type). Additionally, our study included a heterogeneous group including residual, recurrent, and second primary tumors with the first tumor treated by different treatment strategies. Another drawback of this study is the lack of objective postoperative functional assessments for speech and swallowing outcomes. As a result of the multitude of variables, missing data were present for important secondary endpoints, which might bias the results. However, our study resulted in a large amount of 405 patients, which, to our knowledge, is the largest described at the time of writing this manuscript and the included amount of patients largely exceeds the sample size of most other published studies. Thanks to this sample size, a multivariable analysis could be performed, correcting for confounding, and making the reported findings more robust and reliable.

## Conclusion

Substantial complication rates, comparable to the rates described in the literature, and favorable functional outcomes are reported after STL. In a multivariable model, lower preoperative hemoglobin (<12.5 g/dl) was identified as an independent prognostic factor for higher overall complication rate. No risk factors were found significant for clinical fistula formation. Vascularized tissue augmentation did not significantly prevent clinical PCF. However, when clinical and subclinical PCFs were taken together (“overall PCF”), the use of a PM muscle onlay flap was confirmed to have a protective effect while prior radiochemotherapy was shown to have a negative effect on overall PCF rate on multivariable analysis. Patients with positive section margins and patients who developed a PCF or were treated with surgery combined with adjuvant RT (vs. RT alone) for their first tumor were less likely to achieve total peroral intake postoperatively. Postoperative dysphagia proved more likely in patients who developed a PCF, and less likely in patients who underwent STL without partial pharyngectomy and in patients with no or myocutaneous PM flap reconstruction, compared to muscle onlay PM flap. Achieving postoperative functional speech proved most likely in patients with smaller tumors (lower pT classification) and free section margins.

## Data Availability Statement

The raw data supporting the conclusions of this article will be made available by the authors, without undue reservation.

## Ethics Statement

The studies involving human participants were reviewed and approved by University Hospital Leuven Committee for Medical Ethics. Written informed consent for participation was not required for this study in accordance with the national legislation and the institutional requirements.

## Author's Note

Abstract of this paper was accepted for podium presentation during the 13th European Laryngology Society in Stuttgart, Germany (scheduled for June 17-20 2020, but postponed due to the COVID19 pandemic).

## Author Contributions

JM and HD equally contributed to study set-up, data quality control, drafting manuscript, and review of manuscript. JD, GB, TM, and PN contributed to data collection, drafting manuscript, and review of the manuscript. AL contributed to statistical analysis, drafting, and review of manuscript. AG, CV, TV, PD, WH, and VV contributed to study set-up, drafting manuscript, and review of manuscript. All authors contributed to the article and approved the submitted version.

## Conflict of Interest

The authors declare that the research was conducted in the absence of any commercial or financial relationships that could be construed as a potential conflict of interest.

## Abbreviations

Std: Standard deviation
IQR: Interquartile range
CRT: Chemoradiation therapy
RT: Radiation therapy
PM: Pectoralis major
MRND: Modified radical neck dissection
STL: Salvage total laryngectomy
TEP: Tracheo-esophageal puncture
OR: Odds ratio
CI: Confidence interval
TL: Subgroup total laryngectomy
RE: Relative effect.
